# Task Offloading Based on Lyapunov Optimization for MEC-Assisted Vehicular Platooning Networks

**DOI:** 10.3390/s19224974

**Published:** 2019-11-15

**Authors:** Taiping Cui, Yuyu Hu, Bin Shen, Qianbin Chen

**Affiliations:** 1 School of Communication and Information Engineering, Chongqing University of Posts and Telecommunications, Nan-An District, Chongqing 400065, China; shenbin@cqupt.edu.cn; 2 Chongqing Key Labs of Mobile Communications, Chongqing 400065, China; chenqb@cqupt.edu.cn

**Keywords:** mobile edge computing, vehicular platooning, task offloading, Lyapunov optimization

## Abstract

Due to limited computation resources of a vehicle terminal, it is impossible to meet the demands of some applications and services, especially for computation-intensive types, which not only results in computation burden and delay, but also consumes more energy. Mobile edge computing (MEC) is an emerging architecture in which computation and storage services are extended to the edge of a network, which is an advanced technology to support multiple applications and services that requires ultra-low latency. In this paper, a task offloading approach for an MEC-assisted vehicle platooning is proposed, where the Lyapunov optimization algorithm is employed to solve the optimization problem under the condition of stability of task queues. The proposed approach dynamically adjusts the offloading decisions for all tasks according to data parameters of current task, and judge whether it is executed locally, in other platooning member or at an MEC server. The simulation results show that the proposed algorithm can effectively reduce energy consumption of task execution and greatly improve the offloading efficiency compared with the shortest queue waiting time algorithm and the full offloading to an MEC algorithm.

## 1. Introduction

In several typical application scenarios under the fifth-generation (5G) cellular networks, the huge number of intelligent vehicles (2.8 billion) will be a question worth pondering by 2020 [1]. In the 3rd Generation Partnership Project Technical (3GPP) Report [2], 27 use cases of vehicle-to-everything (V2X)were proposed to intend to help drivers in avoiding or mitigating rear-end vehicle collisions in the forward path of travel. Different V2X scenarios require the transport of V2X messages with different performance requirements for the 3GPP system. The Technical Specification [3] specifies service requirements to enhance 3GPP support for V2X scenarios in five areas and vehicle platooning is one of the areas. Platooning, as a vehicular traffic management strategy, is a key step for autonomous driving in intelligent transportation systems (ITS). In general, the platooning consists of two types of members: one is the leader (commander) and the other is the member of platooning (including the relay vehicle and tail vehicle) [4]. Vehicles in platooning run on the same driveway, and the distances between vehicles are approximately the same. In platooning, vehicles use virtual strings to connect adjacent vehicles and control them by updating real-time motion data (such as the driving distance between vehicles and speed). Most vehicle-related tasks, such as automatic drive, 3D navigation, voice processing, traffic information system, etc, are typically computation-intensive tasks that require more computing resources and energy to process [5].

Generally, vehicles have limited computing resources and battery lifetime, bringing great challenges at effectively addressing these computation-intensive mobile applications. A solution based on cloud computing is proposed by the academic community, where the computation data are transmitted to a remote cloud center for execution [6]. However, if such typical computation-intensive tasks are transferred to a remote cloud center, the transmission delay may not be able to meet the requirement of ultra-low delay for vehicular applications and services. The European Telecommunications Standards Institute (ETSI) has proposed a promising paradigm-mobile edge computing (MEC) [7,8], which is an advanced technology to support multiple applications and services that requires ultra-low latency. In an MEC-assisted vehicular network, a mobile application can be executed on the vehicle itself (local execution) or offloaded to an MEC server (edge execution) for processing. Because of the short distance between an MEC server and a vehicle, the MEC paradigm provides high bandwidth, low latency, and computational agility in computation offloading [9].

In this paper, we consider a platooning with an MEC-assisted server to execute computing tasks of vehicle terminals. An optimal offloading decision is investigated among the members of the platooning and MEC server to minimize the average total energy consumption at each time within the tasks’ execution deadlines. Mathematically, the classical Lyapunov algorithm is adopted to simplify the research objective. Then, a sub-optimal solution of the simplified problem is obtained by a greedy algorithm. The simulation results show that the proposed algorithm can effectively reduce the energy consumption of task execution and greatly improve the offloading efficiency of vehicles compared with the shortest queue waiting time algorithm and the full offloading to an MEC algorithm.

The rest of this paper is organized as follows. We review related work in Section 2. In Section 3, we present the system model. In Section 4, we describe the formulation of our optimization problem. A solution is provided in Section 5. Section 6 shows the simulation results, and Section 7 concludes the paper.

## 2. Related Work

Since the concept of MEC was proposed, experts and scholars have conducted in-depth research on it [10]. The key technologies of MEC mainly include task offloading technology, wireless data caching technology, and local offloading technology based on a software-defined network (SDN), among which MEC offloading technology is an important approach for the MEC system to realize real-time processing of terminal services [11]. Currently, task offloading assisted by MEC has been attracted by a lot of researchers. Among this research, energy consumption is one of the most concerned issues. Researchers consider maximum delay, computing resources, channel resources, power allocation, interference, etc. as optimization constraints, to minimize energy consumption [12,13,14,15], to minimize cost [16,17,18], or to maximize server revenue [19,20,21], etc. References [12,14,15,16] aimed at reducing the energy consumption of MEC system. The authors in [12,14] jointly optimized offloading decisions, wireless resource allocation, and computation resource allocation, for which [12] designed a heuristic scheme to minimize energy consumption of mobile devices. In [13], Zhao et al. proposed a branched-bound (RLTBB) method based on linearization technology to obtain the minimum energy consumption, which can obtain the optimal or sub-optimal results by solving the precision. The authors considered the trade-off between energy consumption and delay, and proposed a power minimization problem based on the stability constraint of the task buffer [14]. In [15], an efficient and energy-saving offloading decision algorithm based on Lyapunov optimization was proposed, which could minimize the average energy consumption of mobile devices under the premise of satisfying the delay constraint. In addition, a Lyapunov algorithm can significantly reduce energy consumption at the expense of only a small portion of response time compared to local and remote execution, and it can not only optimize the energy more effectively, but also reduce the computational complexity compared with Lagrangian relaxation algorithm.

The vehicle platooning were investigated in [5,22,23]. In [5], Wang et al. proposed a platoon communication mode based on D2D technology to improve the stability and efficiency of vehicle platooning with limited spectrum resources. In addition, then, a two-stage platoon formation algorithm based on platoon leader evaluation mechanism was proposed to form stable platoons. Several key issues need to be solved in the coordinated adaptive cruise control of human and autonomous driving, such as the difficulty to adaptively control the vehicle speed and distance adjacent vehicles. In the solving of these problems, the author [22] proposed a collaborative adaptive driving vehicle cloud computing method based on mobile edge computing, which effectively avoids the shock wave generated when driving in a platoon. The authors investigated a task offloading approach for an MEC-assisted vehicle platooning, where the Lyapunov optimization algorithm is employed for solving the optimization problem under the condition of stability of task queues [24]. In [23], Fan et al. studied the offloading decision of cooperative processing tasks between platooning and the MEC server. The authors transformed the task decision-making problem with minimum cost into the shortest path problem, and uses the Lagrange relaxation algorithm to solve the problem approximately.

Thus far, there is still a lack of research on how to offload tasks on vehicle platooning. An MEC-assisted vehicle platooning is investigated, and each vehicle member has the ability to handle other offloading tasks. The optimal offloading strategy is achieved through the Lyapunov optimization algorithm to minimize the average total energy consumption each time. The contributions of this paper are summarized as follows:

- The problem of task offloading optimization is modeled as a minimum average total energy consumption each time by combining each member in platooning with the MEC server.
- To meet the requirements of the execution deadline and energy consumption, an optimization algorithm based on Lyapunov function is proposed, and a greedy algorithm is adopted to approximate the sub-optimal decision.
- The proposed algorithm can significantly reduce the energy consumption of platooning members (PMs) compared with the shortest queue waiting time algorithm and the full offloading to an MEC algorithm.

## 3. System Model

In this section, we first present a system model, including the task offloading scenario, task offloading system model and task offloading queue model. Then, the communication model and computation model are provided. The key notations used in this paper are summarized in Table 1.

### 3.1. Task Offloading Model

**Figure 1:**
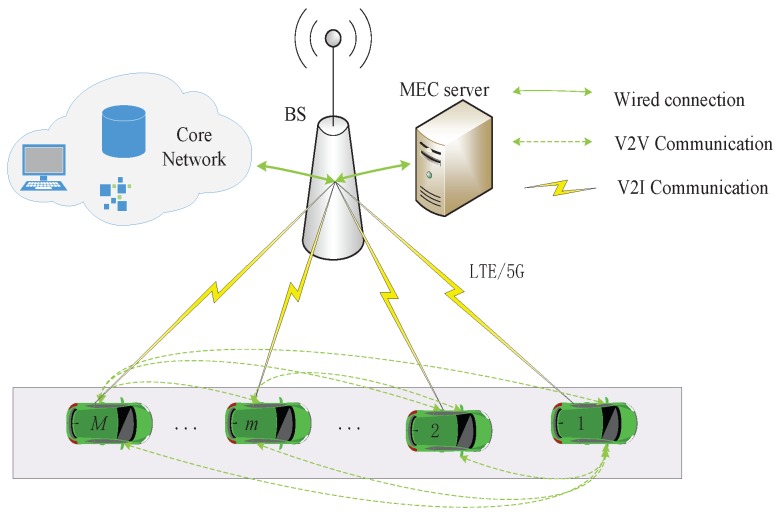
Task offloading scenario.

Figure 1 depicts the task offloading scenario in this paper, where vehicle-to-vehicle (V2V) communication is adopted between vehicles, and vehicle-to-infrastructure (V2I) communication is adopted between vehicle and base station (BS) [25,26]. A cellular network can provide sufficiently stable/reliable wireless communication for V2V and V2I communication. In three application scenarios under the 5G cellular network, URLLC (Ultra-Reliable and Low Latency Communications) not only guarantees ultra-low transmission delay, but also guarantees ultra-reliability transmission [27]. Without loss of generality, it is assumed that the entire platooning is covered by a cellular network, and all PMs and MEC can directly communicate in pairs. There are *M* PMs in the platooning. Starting from the driving direction of the platooning, the first member is defined as 1, and then the serial numbers of other PMs increase consecutively. An MEC server is deployed with a BS via wired connection [28,29,30,31]. If a vehicle sends information to an MEC server, it first should send information to BS through V2I, and then forward to the MEC server through wired transmission.

**Figure 2:**
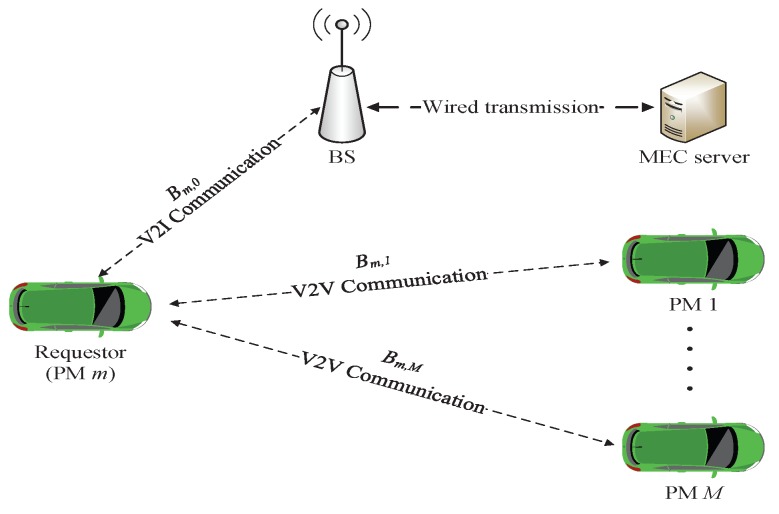
Task offloading system model.

Figure 2 illustrates the task offloading system model. The task requester in platooning is denoted as m∈{1,2,…,M}, and the computation node of the task is denoted as k∈{0,1,…,M}, in which 0 represents the MEC server. $B_{m,k}$ is the transmission rate between PM *m* and computation node *k*. When m=k, the task is executed at the local *m*. Due to the fast transmission rate of wired transmission and the co-existing deployment between the BS and MEC server, the wired transmission time is ignored in this paper [28].

**Figure 3:**
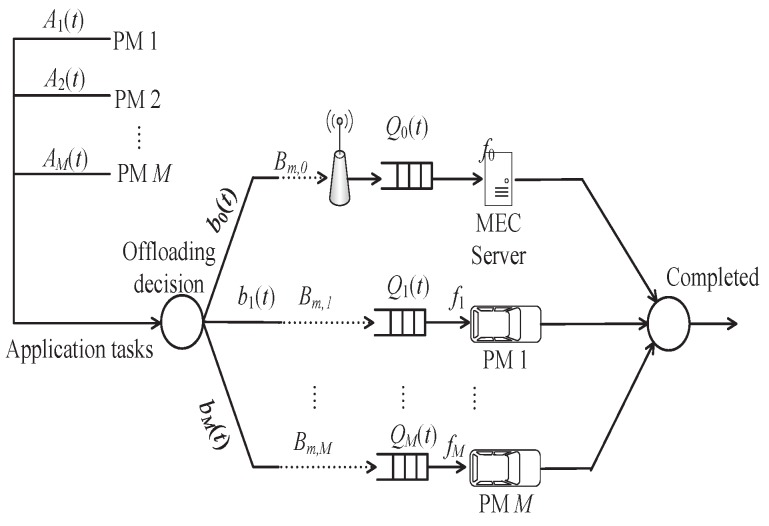
Task offloading queue model.

All vehicles in platooning will generate a computation task in each time interval, that is, all PMs are task requesters. Notice that there are a total of *M* PMs in platooning. Thus, each PM has a total of M+1 computation nodes, namely *M* PMs and an MEC server. In addition, the time of offloading task is discretized, and time *t* is to represent the execution slot of the task. Then, the offloading member should transfer the offloading tasks to other PMs or the MEC server for execution through V2V or V2I. The task offloading queue model is shown in Figure 3, where $Q_{k}\left(t\right)\in \left\{Q_{0}\left(t\right),Q_{1}\left(t\right),\ldots ,Q_{M}\left(t\right)\right\}$ represents the length of task queue *k* at time *t*. The task load arrived at PM *m* in time *t* is $A_{m}\left(t\right)$, which is expressed by the number of CPU cycles required to execute the task. $A_{m}\left(t\right)\in \left\{A_{1}\left(t\right),A_{2}\left(t\right),\ldots ,A_{M}\left(t\right)\right\}$ follows Poisson distribution with mean $℧\left\{A_{m}\left(t\right)\right\}=\lambda_{m}$, and satisfies independent identical distribution. $b_{k}\left(t\right)\in \left\{b_{0}\left(t\right),b_{1}\left(t\right),\ldots ,b_{M}\left(t\right)\right\}$ represents the task computation workload that arrives at queue *k* after offloading decision at time *t*. For instance, when PM 1 and PM 2 decide to offload the task to MEC server at time *t*, $b_{0}\left(t\right)=A_{1}\left(t\right)+A_{2}\left(t\right)$ can be obtained. Each vehicle can provide a certain amount of computation resources [28,32,33], and $f_{k}\in \left\{f_{0},f_{1},\ldots ,f_{M}\right\}$ represents the computation capacity (i.e., CPU cycles per second) provided by the computation node *k* in each time interval.

### 3.2. Communication Model

In this vehicular network, the path loss models between vehicles [34] and between vehicle and BS [35] are computed by

(1) $$PL\left(l_{m,k}\right)=\left\{\begin{array}{cc} 63.3+17.7lg(l_{m,k}) & k\neq 0,\\ 128.1+37.5lg(l_{m,k}) & k=0, \end{array}\right.$$

where $l_{m,k}$ represents the distance between vehicle *m* and computation node *k*. Then, the corresponding data transmission rate can be calculated by

(2) $$B_{m,k}\left(t\right)=\left\{\begin{array}{cc} w_{1}log_{2}\left(1+\frac{\zeta_{v}PL\left(l_{m,k}\right)}{N_{0}}\right) & k\neq 0,\\ w_{2}log_{2}\left(1+\frac{\zeta_{g}PL\left(l_{m,k}\right)}{N_{0}}\right) & k=0, \end{array}\right.$$

where $w_{1}$ and $w_{2}$ are the transmission bandwidths between vehicles and between vehicle and BS, respectively. $N_{0}$ is the noise power, $\zeta_{v}$ and $\zeta_{g}$ are the transmission power of PM and BS, respectively.

### 3.3. Computation Model

The task completion time when the task is offloaded from PM *m* to node *k* at time *t* can be expressed as

(3) $$\begin{array}{c} T_{m}^{k}\left(t\right)\in \left\{T_{m}^{0}\left(t\right),T_{m}^{1}\left(t\right),\ldots ,T_{m}^{M}\left(t\right)\right\}, \end{array}$$

where $T_{m}^{k}\left(t\right)$ includes task transmission time and execution time, which can be computed by

(4) $$T_{m}^{k}\left(t\right)=\left\{\begin{array}{cc} \frac{D_{m}\left(t\right)}{B_{m,k}}+\frac{A_{m}\left(t\right)}{f_{k}} & m\neq k,\\ \frac{A_{m}\left(t\right)}{f_{k}} & m=k, \end{array}\right.$$

where m=k represents that the task is executed locally, that is, there is no transmission delay, while m≠k indicates that the task is offloaded to other node. $D_{m}\left(t\right)\in \left\{D_{1}\left(t\right),D_{2}\left(t\right),\ldots ,D_{M}\left(t\right)\right\}$ denotes the size of data transmitted by PM *m* at time *t*, which is uniformly distributed and independent.

Similarly, the energy consumption is denoted by

(5) $$E_{m}^{k}\left(t\right)\in \left\{E_{m}^{0}\left(t\right),E_{m}^{1}\left(t\right),\ldots ,E_{m}^{M}\left(t\right)\right\},$$

where $E_{m}^{k}\left(t\right)$ represents the energy consumption of PM *m* when offloading the task to queue *k* at time *t*. Then, we obtain

(6) $$E_{m}^{k}\left(t\right)=\left\{\begin{array}{cc} p_{tr}\cdot \frac{D_{m}\left(t\right)}{B_{m,k}}+p_{i}\cdot \frac{A_{m}\left(t\right)}{f_{k}} & m\neq k,\\ p_{c}\cdot \frac{A_{m}\left(t\right)}{f_{k}} & m=k, \end{array}\right.$$

where m=k indicates that the task is executed locally, and m≠k means that the task is offloaded. $P_{tr}$ represents the transmission power of a vehicle, $p_{i}$ represents the idle power of a vehicle, and $p_{c}$ represents the computation power when the task is executed locally.

## 4. Problem Formulation

In this paper, energy consumption is considered from the perspective of a task requester [13,36,37]. The objective of the optimization is to obtain an efficient offloading strategy that enables all PMs to complete tasks with minimal average total energy consumption. Therefore, the optimization problem can be formulated as

(7) $$\begin{array}{cc} \; & \min\bar{E}\overset{\Delta }{=}\lim_{t\to +\infty }\sup\frac{1}{t}\sum_{\tau =1}^{t}\sum_{m=1}^{M}℧\left\{E_{m}\left(\tau \right)\right\},\\ s.t.\; & C1:\bar{Q}\overset{\Delta }{=}\lim_{t\to +\infty }\sup\frac{1}{t}\sum_{\tau =1}^{t}\sum_{k=0}^{M}℧\left\{Q_{k}\left(\tau \right)\right\}<\infty ,\; \end{array}$$

where ℧ is the sign for averaging, and $\bar{E}$ represents the average energy consumption of the task. The constraint condition means that all queues are completed under convergence, where $\bar{Q}$ represents the average queue length of tasks.

## 5. Problem Solution

The optimal offloading decisions are obtained by employing a Lyapunov algorithm and greedy algorithm under the condition of ensuring the stability of task computation queue and meeting the deadline of task execution.

### 5.1. Offloading Decision

The offloading decision vector at time *t* is expressed as

(8) $$a\left(t\right)=[a_{m}\left(t\right)|m\in \left\{1,2,\ldots M\right\},a_{m}\left(t\right)\in \left\{0,1,\ldots M\right\}],$$

where $a_{m}\left(t\right)=k$ means that PM *m* offloads the task to computation node *k* for execution, and we can also express it as $a_{m,k}\left(t\right)=1$. In particular, when m=k, it means that the task is executed locally. Therefore, the offloading decision of PM *m* can be expressed as

(9) $$\begin{array}{c} \left\{\begin{array}{c} a_{m,k}\left(t\right)=1,\\ \sum_{k=0}^{M}a_{m,k}\left(t\right)=1, \end{array}\right. \end{array}$$

where the below equation is to ensure that a task can only be processed at one computing node.

$b_{k}\left(t\right)$ represents the computation size of the task that reaches the *k*-th queue at time *t*, which is related to the offloading decision. Then,

(10) $$b_{k}\left(t\right)=\sum_{m=1}^{M}a_{m,k}\left(t\right)\cdot A_{m}\left(t\right).$$

$T\left(a\left(t\right)\right)=\left\{T_{1}\left(t\right),T_{2}\left(t\right),\ldots ,T_{M}\left(t\right)\right\}$ is the set of execution length for all PMs at time *t*, and it can be expressed by

(11) $$T\left(a\left(t\right)\right)=\left\{\sum_{k=0}^{M}a_{1,k}\left(t\right)\cdot T_{1}^{k}\left(t\right),\sum_{k=0}^{M}a_{2,k}\left(t\right)\cdot T_{2}^{k}\left(t\right),\ldots ,\sum_{k=0}^{M}a_{M,k}\left(t\right)\cdot T_{M}^{k}\left(t\right)\right\},$$

where $T_{m}^{k}\left(t\right)$ is obtained by Equation (4).

Therefore, the total energy consumption also can be expressed as

(12) $$E\left(a\left(t\right)\right)=\sum_{m=1}^{M}\sum_{k=0}^{M}a_{m,k}\left(t\right)\cdot E_{m}^{k}\left(t\right),$$

where $E_{m}^{k}\left(t\right)$ is obtained by Equation (6).

### 5.2. Optimization Based on Lyapunov

An offloading decision algorithm based on Lyapunov optimization theory is proposed in the paper to jointly optimize energy consumption and queue waiting time on the premise of ensuring the stability of the task computation queue, and finally minimizes the task average total energy consumption.

The dynamic queue length at time t+1 is expressed as

(13) $$Q_{k}\left(t+1\right)=\max\left[Q_{k}\left(t\right)-f_{k},0\right]+b_{k}\left(t\right).$$

Before further discussion on the offloading strategy, Lemma 1 related to the derivation of the decision function is provided.

**Lemma** **1.** *Assuming that X, Y, Z, and W are non-negative positive real number and X=max[Y−Z,0]+W, $X^{2}\leq Y^{2}+W^{2}+Z^{2}-2Y\left(Z-W\right)$ can be obtained.*

Based on Lyapunov optimization theory, the Lyapunov function is expressed as

(14) $$L\left(t\right)=\frac{1}{2}\sum_{k=0}^{M}Q_{k}^{2}\left(t\right)$$

According to Lemma 1, the following equation

(15) $$\begin{array}{cc} L(t+1)-L(t) & =\frac{1}{2}\sum_{k=0}^{M}\left[Q_{k}^{2}\left(t+1\right)-Q_{k}^{2}\left(t\right)\right]\\ \; & =\frac{1}{2}\sum_{k=0}^{M}{\left[\max\left[Q_{k}\left(t\right)-f_{k},0\right]+b_{k}\left(t\right)\right]}^{2}-Q_{k}^{2}\left(t\right)\\ \; & \leq \frac{1}{2}\sum_{k=0}^{M}\left(f_{k}^{2}+b_{k}^{2}\left(t\right)\right)+\sum_{k=0}^{M}Q\left(t\right)\cdot \left[b_{k}\left(t\right)-f_{k}\left(t\right)\right] \end{array}$$

can be obtained from Equation (14).

Then, the Lyapunov transfer function is expressed as

(16) Δ(t)=E{L(t+1)−L(t)|Q(t)}.

Inequality (15) is substituted for Equation (16) to obtain

(17) $$\Delta \left(t\right)\leq \frac{1}{2}\sum_{k=0}^{M}E\{\left(f_{k}^{2}+b_{k}^{2}\left(t\right)|Q\left(t\right)\right\}-\sum_{k=0}^{M}E\left\{Q_{k}\left(t\right)f_{k}\left(t\right)|Q\left(t\right)\right\}+\sum_{k=0}^{M}E\left\{Q_{k}\left(t\right)b_{k}\left(t\right)|Q\left(t\right)\right\}.$$

According to the above Lyapunov optimization method, it is necessary to ensure the stability of the queue while minimizing the penalty term. The Lyapunov penalty term in this paper refers to the energy consumption of the task performed, namely ℧{E(a(t))|Q(t)}. The Lyapunov transfer penalty term is

(18) Δ(t)+V℧{E(a(t))|Q(t)},

where *V* is a weight control parameter, which indicates the weight of minimizing energy consumption. In other words, *V* can be considered as a threshold of the system state. According to Equations (17) and (18), we can deduce

(19) $$\begin{array}{cc} \Delta (t)+V℧\{E(a(t))|Q(t)\} & \leq \frac{1}{2}\sum_{k=0}^{M}℧\{\left(f_{k}^{2}+b_{k}^{2}\left(t\right)|Q\left(t\right)\right\}\\ +V℧\{E(a(t))|Q(t)\} & -\sum_{k=0}^{M}℧\left\{Q_{k}\left(t\right)b_{k}\left(t\right)|Q\left(t\right)\right\}\\ +\sum_{k=0}^{M}℧\left\{Q_{k}\left(t\right)b_{k}\left(t\right)|Q\left(t\right)\right\} & =\frac{1}{2}\sum_{k=0}^{M}f_{k}^{2}-\sum_{k=0}^{M}Q_{k}\left(t\right)f_{k}\\ +℧\{[\frac{1}{2}\sum_{k=0}^{M}(\sum_{m=1}^{M}a_{m,k} & \left(t\right)\cdot A_{m}{\left(t\right))}^{2}+VE\left(a\left(t\right)\right)\\ +\sum_{k=0}^{M}\sum_{m=1}^{M}Q_{m,k}\left(t\right)\cdot & a_{m,k}\left(t\right)\cdot A_{m}\left(t\right)]|Q\left(t\right)\}. \end{array}$$

### 5.3. Optimization Based on a Greedy Algorithm

To achieve the objective of minimizing the average total energy consumption, the appropriate offloading decision should be found first. When minimizing the right side of Inequality (19), minimizing energy consumption while satisfying queue stability can be obtained. Ignoring the constant term on the right side of Inequality (19), the optimization problem can be formulated as

(20) $$\begin{array}{cc} \arg\min_{a(t)} & \frac{1}{2}\sum_{k=0}^{M}{\left(\sum_{m=1}^{M}a_{m,k}\left(t\right)\cdot A_{m}\left(t\right)\right)}^{2}+VE\left(a\left(t\right)\right)\\ \; & +\sum_{k=0}^{M}\sum_{m=1}^{M}Q_{m,k}\left(t\right)\cdot a_{m,k}\left(t\right)\cdot A_{m}\left(t\right)\\ s.t.\;\; & C2:\frac{D_{m}\left(t\right)}{B_{m,k}\left(t\right)}+\frac{\max\left\{Q_{k}\left(t\right)-f_{k},0\right\}+b_{k}\left(t\right)}{f_{k}}\leq T_{d},\\ \; & \;m\in \{1,2,\ldots ,M\},k\in \{0,1,\ldots ,M\}\\ \; & C3:f_{0}>f_{m}>0\\ \; & C4:a_{m,k}\left(t\right)\in \left\{0,1\right\}\\ \; & C5:\sum_{k=0}^{M}a_{m,k}\left(t\right)=1, \end{array}$$

where C2 is to ensure that the task can be completed within the deadline. C3 refers to the computation resources provided by an MEC server being always greater than those provided by other PMs. C4 indicates that the status of the offloading decision is 0 or 1, and C5 means that a task can only be offloaded to one PM or MEC server. Definitely, the amount of computation may not be within the very specific range. In this case, the amounts of demanded computing resources are beyond the capability of the platooning and MEC. In other words, the delay/deadline constraint C2 cannot be met due to the limited computing resources that the platoon and MEC can provide, and the execution cannot be completed before the task deadline.

Notice that each PM in this paper has *M* + 1 possible options. In order to obtain the optimal solution, it is necessary to repeat iterations ${\left(M+1\right)}^{M}$ times. This is a knapsack problem, and the problem scale is quite large with the increase of *M*. The greedy algorithm is adopted to approximately solve the optimization problem as shown in Algorithm 1 [38].

**Algorithm 1** Greedy Algorithm
1:Initialization: set Formulation (20) as $Y(a_{mk}\left(t\right))$, let *Z* be a variable  2:
**for** m=1:M **do**
3: $Z=Y(a_{m0}\left(t\right))$  4: **for** k=0:M **do**
5:  **if** $Y(a_{mk}\left(t\right))<Z$ **then**
6:   $Z=Y(a_{mk}\left(t\right))$  7:  **else**8:   **CONTINUE**  9:  **end if** 10:  $k^{*}{=argZ|}_{k}$  11:  **return** $a_{m}\left(t\right)=k^{*}$12: **end for**13:**end for** 


In Algorithm 1, we set the value of Formulation (20) as $Y(a_{mk}\left(t\right))$ first, and each vehicle finds its own offloading node with the smallest $Y(a_{mk}\left(t\right))$ value as the optimal decision through iteration of all offloading nodes. In this way, a better offloading decision can be obtained while greatly reducing the complexity of the algorithm.

## 6. Simulation Results

In this section, we first set the main parameters, and then present the simulation results to estimate the performance of the optimization scheme.

### 6.1. Parameter Settings

The platooning consists of five members, namely *M* = 5. The computation resources of MEC server and PMs are $f_{0}=1500$ Hz, $f_{1}=200$ Hz, $f_{2}=650$ Hz, $f_{3}=250$ Hz, $f_{4}=500$ Hz, $f_{5}=850$ Hz, respectively. The average number of CPU cycles consumed by each vehicle to calculate its own task is $\lambda_{1}=45$ cycles, $\lambda_{2}=60$ cycles, $\lambda_{3}=100$ cycles, $\lambda_{4}=20$ cycles, $\lambda_{5}=80$ cycles, respectively. The data transmission power of all PMs is $p_{tr}$ = 0.1 W [39,40], computation power is $p_{c}$ = 0.5 W [41], and idle power is $p_{i}$ = 0.001 W. The distance is the same between adjacent vehicles. Each time interval of simulation is set as 0.1 s, and the total simulation time is 30 s. All simulation parameters are summarized in Table 2.

### 6.2. Performance Analysis

**Figure 4:**
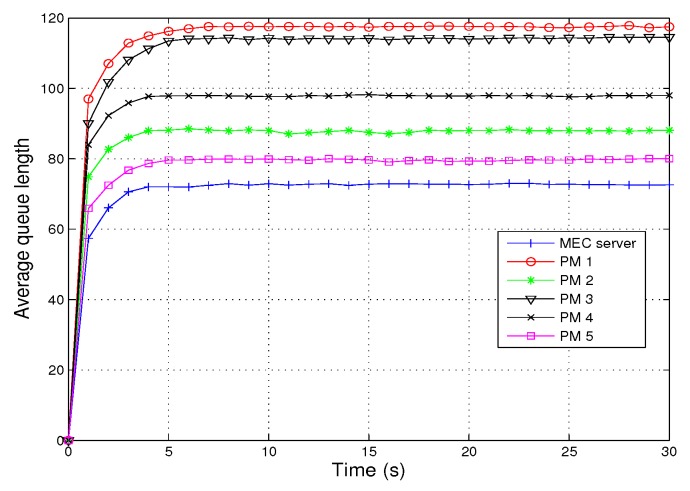
Average queue length vs. time.

Figure 4 shows the relationship between average queue length and time. From the simulation results, it can be seen that the average length of each queue increases rapidly with time, then slowly tends to be flat until it reaches a stable state. The queue length is related to the size of computation resources corresponding to queue, and the larger the computation resources provided, the smaller the queue length.

**Figure 5:**
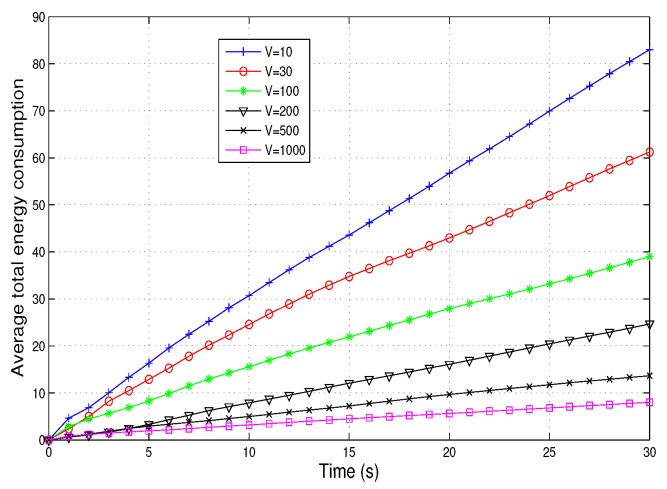
Average total energy consumption vs. time.

Figure 5 shows the variation of average energy consumption with time. *V* is an important parameter to control the energy consumption of the whole system. As the value of *V* increases, the energy consumption decreases because *V* indicates the importance attached to energy consumption. According to the optimization function Formulation (20), the larger the value of *V*, the greater the proportion of energy consumption in the entire optimization function. In addition, we set the value of *V* to 1000 in this system, which is an optimal value. Further increasing the value of *V* will not affect the energy consumption because the proportion of energy consumption in the whole optimization function is much larger than other items.

**Figure 6:**
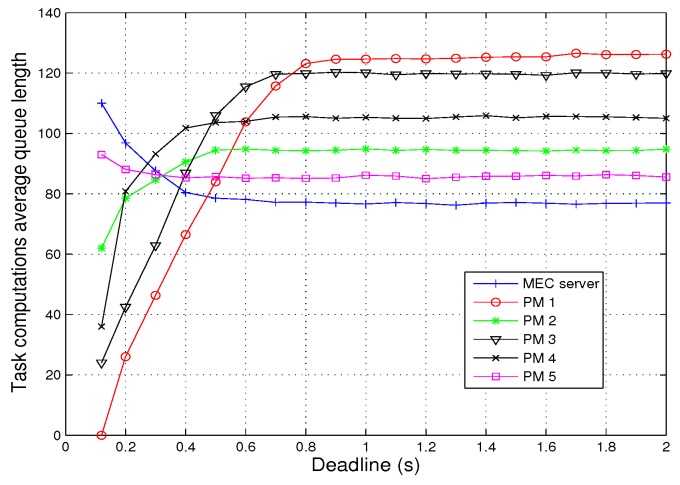
Task computation average queue length vs. deadline.

Figure 6 shows the effect of deadline $T_{d}$ on average queue length of task computation. The minimum deadline that the system can meet is 0.12 s. If the deadline is less than 0.12 s, some tasks cannot be completed. When 0.12 s < $T_{d}$ < 0.9 s, some tasks will be offloaded to another queue to ensure the completion of the tasks because the resources of the PM cannot meet its task requirements. When $T_{d}$ > 0.9 s, the queue length will not change with $T_{d}$ because all tasks can be completed within 0.9 s. In addition, the queues of MEC server and PM 5 show a downward trend because the computation resources they provide are large enough, and these two queues can meet more task offloading requirements. However, due to the smaller computational resources provided by other queues, fewer and fewer tasks are offloaded to PM 1, 2, 3, and 4 in order to meet the offloading demand as the deadline $T_{d}$ falls continuously. Therefore, the average queue length of their task computation size will be shorter and shorter as $T_{d}$ decreases.

**Figure 7:**
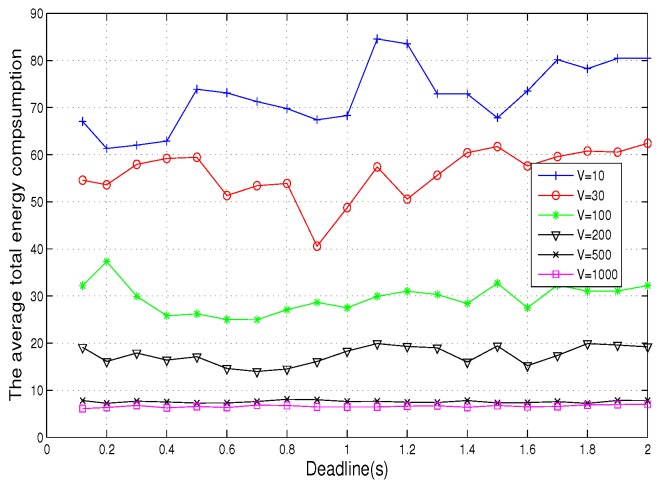
Average total energy consumption vs. deadline.

In Figure 7, the energy consumption fluctuates greatly with $T_{d}$ when the value *V* is small, while, when the value of *V* is large, the fluctuation of energy consumption is relatively stable. Because in the optimization function Formulation (20), when *V* = 10, the value of the whole penalty item is about 0.01 times of the value of other items, so the proportion of the whole penalty item is relatively small, and the fluctuation is also relatively large. However, when *V* = 1000, the penalty term accounts for the vast majority of the entire optimization function, and the energy consumption changes dominate, so the energy consumption fluctuation tends to be stable when the *V* value is large enough.

To verify the effectiveness of the proposed algorithm, it is compared with the following two schemes: (1) the shortest queue waiting time algorithm, where $t_{d}^{k}=Q_{k}/f_{k}$ indicates the waiting time of the queue *k*, and the shortest queue waiting time algorithm indicates that the minimum value of all queue waiting times is treated as the deadline of all tasks; (2) all tasks are offloaded to the MEC server for execution.

**Figure 8:**
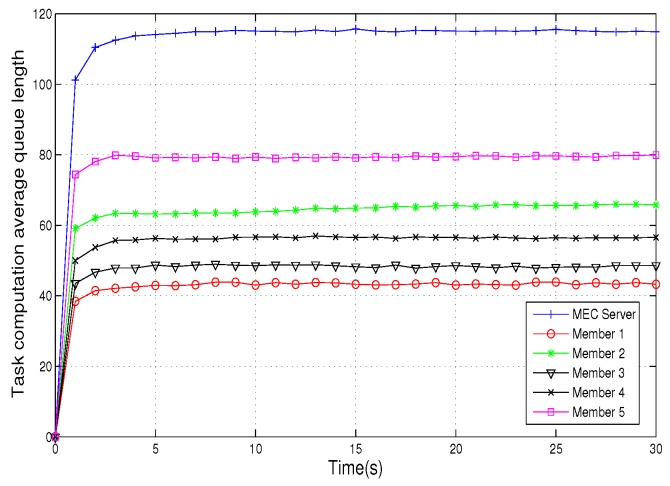
Task computation average queue length vs. time.

Figure 8 shows the trend of the task queue length of the shortest queue waiting time algorithm with time. The figure shows that the MEC server has the longest queue because the MEC server provides the maximum computation resources, so the wait time is shorter than other PMs, and more tasks will be offloaded to the MEC server. PM 1, on the other hand, provides the minimal computation resources, so the queue has the longest wait times, and fewer tasks are offloaded to PM 1.

**Figure 9:**
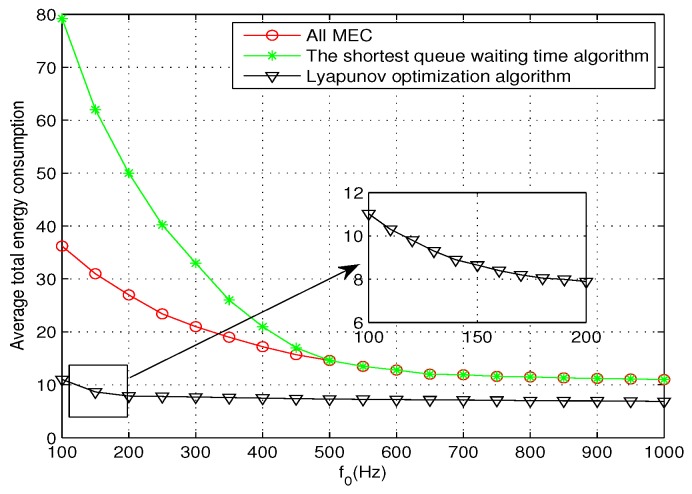
Performance comparison diagram.

Figure 9 shows a performance comparison of the three methods, where the small graph shows the change of energy consumption by using the optimization algorithm when the frequency is from 100 to 200 Hz. As can be seen from the graph, the proposed algorithm is superior to the other two schemes. Firstly, for the shortest queue waiting time algorithm, there will be some tasks with a large computation load offloaded to PMs with less computational resources, resulting in excessive computation energy consumption. Secondly, for the tasks with less computation and larger data transmission, offloading to PMs can save energy better than offloading to a MEC server. In addition, the two comparison algorithms overlap after $f_{0}=500$ Hz because, when $f_{0}$ increases, the waiting time of MEC server decreases gradually, and more tasks are offloaded to the MEC server. When $f_{0}$ is large enough, all tasks can be computed directly without waiting. The algorithm in this paper will automatically adjust the offloading location according to the task parameters to optimize the energy consumption, which is the reason why the algorithm is superior to the other two schemes.

## 7. Conclusions

The task offloading for a vehicular platooning assisted by an MEC server was investigated in this paper. An optimized task offloading algorithm was proposed to reduce the average total energy consumption while meeting the deadline of tasks. In this paper, the targeted problem is simplified by the Lyapunov optimization method, and the simplified problem is approximately solved by a greedy algorithm. Thus, the sub-optimal task offloading decision is obtained. By setting a reasonable control parameter *V* for the Lyapunov optimization function, energy consumption takes a decisive proportion in the whole optimization function. In addition, the proposed algorithms can not only ensure the stability of the task computation queue, but also dynamically adjust the task offloading strategy according to the amount of the task data. It is one of the effective and feasible ways to realize the task dynamic offloading.

## Figures and Tables

**Table 1:** Summary of key notations.

Notation	Definition
*m*	PM ID
*k*	Node ID, including MEC server
*t*	Time *t*
*M*	The number of PM
$Q_{k}\left(t\right)$	Queue k of vehicle or MEC in time *t*
$A_{m}\left(t\right)$	Workload of PM *m* in time *t*
$b_{k}\left(t\right)$	The size of task that arrives at queue *k* in time *t*
$f_{k}$	Computation capability of computing node *k*
$D_{m}\left(t\right)$	Input data of PM *m* at time *t*
$B_{m,k}$	transmission rate between PM *m* and node *k*
$T_{m}^{k}\left(t\right)$	Execution time of task *m* on node *k*
$E_{m}^{k}\left(t\right)$	Energy consumption of task *m* on node *k*
$l_{m,o}$	Positional distance between vehicle *m* and BS
$l_{m,n}$	Positional distance between vehicles
$w_{1}$	Transmission bandwidth between vehicles
$w_{2}$	Transmission bandwidth between vehicle and BS
*V*	A weight control parameter
$P_{tr}$	Data transmission power of vehicle
$P_{c}$	Computation power of vehicle
$P_{i}$	Idle power of vehicles
$N_{0}$	Noise power

**Table 2:** Simulation parameters.

Parameter Name	Value
Number of PMs	*M* = 5
Data transmission power	$p_{tr}=0.1$ W
Computation power	$p_{c}=0.5$ W
Idle power	$p_{i}$ = 0.001 W
The distance between adjacent vehicles	8 m
V2V communication bandwidth	$w_{1}=100$ MHz
V2I communication bandwidth	$w_{2}=20$ MHz
Noise power density	−174 dBm/Hz

## References

[1] Makinen O. Streaming at the Edge: Local Service Concepts Utilizing Mobile Edge Computing. Proceedings of the 2015 9th International Conference on Next, Generation Mobile Applications, Services and Technologies.

[2] 3GPP TR 22.885 V14.0.0 Study on LTE Support for Vehicle to Everything (V2X) Services. https://portal.3gpp.org/desktopmodules/Specifications/SpecificationDetails.aspx?specificationId=2898.

[3] 3GPP TS 22.186 V15.0.0 Enhancement of 3GPP Support for V2X Scenarios. https://portal.3gpp.org/desktopmodules/Specifications/SpecificationDetails.aspx?specificationId=3180.

[4] Axelsson J. (2017). Safety in Vehicle Platooning: A Systematic Literature Review. IEEE Trans. Intell. Transp. Syst..

[5] Wang R., Wu J., Yan J. (2018). Resource Allocation for D2D-Enabled Communications in Vehicle Platooning. IEEE Access.

[6] Mao Y., You C., Zhang J., Huang K. (2017). A Survey on Mobile Edge Computing: The Communication Perspective. IEEE Commun. Surv. Tutor..

[7] Cicirelli F. (2018). Edge Computing and Social Internet of Things for Large-Scale Smart Environments Development. IEEE Internet Things J..

[8] Li H., Shou G., Hu Y., Guo Z. Mobile Edge Computing: Progress and Challenges. Proceedings of the 2016 4th IEEE International Conference on Mobile Cloud Computing, Services, and Engineering (MobileCloud).

[9] Tran T., Pompili D. (2019). Joint Task Offloading and Resource Allocation for Multi-Server Mobile-Edge Computing Networks. IEEE Trans. Veh. Technol..

[10] Wang Y., Sheng M., Wang X., Wang L., Li J. (2016). Mobile-Edge Computing: Partial Computation Offloading Using Dynamic Voltage Scaling. IEEE Trans. Commun..

[11] Taleb T., Samdanis K., Mada B., Flinck H. (2017). Dutta, S. Sabella, D. On Multi-Access Edge Computing: A Survey of the Emerging 5G Network Edge Cloud Architecture and Orchestration. IEEE Commun. Surv. Tutor..

[12] Yu Y., Zhang J., Letaief K. Joint Subcarrier and CPU Time Allocation for Mobile Edge Computing. Proceedings of the 2016 IEEE Global Communications Conference (GLOBECOM).

[13] Zhao P., Tian H., Qin C., Nie G. (2017). Energy-Saving Offloading by Jointly Allocating Radio and Computational Resources for Mobile Edge Computing. IEEE Access.

[14] Mao Y., Zhang J., Song S., Letaief K. Power-Delay Tradeoff in Multi-User Mobile-Edge Computing Systems. Proceedings of the 2016 IEEE Global Communications Conference (GLOBECOM).

[15] Wu H., Sun Y., Wolter K. (2018). Energy-Efficient Decision Making for Mobile Cloud Offloading. IEEE Trans. Cloud Comput..

[16] Chen X., Jiao L., Li W., Fu X. (2016). Efficient Multi-User Computation Offloading for Mobile-Edge Cloud Computing. IEEE/ACM Trans. Netw..

[17] Li J., Gao H., Lv T., Lu Y. Deep reinforcement learning based computation offloading and resource allocation for MEC. Proceedings of the 2018 IEEE Wireless Communications and Networking Conference (WCNC).

[18] Zhang J. (2018). Energy-Latency Tradeoff for Energy-Aware Offloading in Mobile Edge Computing Networks. IEEE Internet Things J..

[19] Liu M., Liu Y. (2018). Price-Based Distributed Offloading for Mobile-Edge Computing With Computation Capacity Constraints. IEEE Wirel. Commun. Lett..

[20] Zhang K., Mao Y., Leng S., Vinel A., Zhang Y. Delay constrained offloading for Mobile Edge Computing in cloud-enabled vehicular networks. Proceedings of the 2016 8th International Workshop on Resilient Networks Design and Modeling (RNDM).

[21] Zhang K., Mao Y., Leng S., Maharjan S., Zhang Y. Optimal delay constrained offloading for vehicular edge computing networks. Proceedings of the 2017 IEEE International Conference on Communications (ICC).

[22] Huang R., Chang B., Tsai Y., Liang Y. Mobile Edge Computing-Based Vehicular Cloud of Cooperative Adaptive Driving for Platooning Autonomous Self Driving. Proceedings of the 2017 IEEE 7th International Symposium on Cloud and Service Computing (SC2).

[23] Fan X., Cui T., Cao C., Chen Q., Kwak K. (2019). Minimum-Cost Offloading for Collaborative Task Execution of MEC-Assisted Platooning. Sensors.

[24] Hu Y., Cui T., Huang X., Chen Q. Task Offloading Based on Lyapunov Optimization for MEC-assisted Platooning. Proceedings of the WCSP 2019.

[25] Jia Q. (2019). Energy-efficient computation offloading in 5G cellular networks with edge computing and D2D communications. IET Commun..

[26] Lozano D., Mateo S. (2019). Review on V2X, I2X, and P2X Communications and Their Applications: A Comprehensive Analysis over Time. Sensors.

[27] Popovski P. (2014). Ultra-reliable communication in 5G wireless systems. Comput. Sci..

[28] Zhao J., Li Q., Gong Y., Zhang K. (2019). Computation Offloading and Resource Allocation For Cloud Assisted Mobile Edge Computing in Vehicular Networks. IEEE Trans. Veh. Technol..

[29] Zhang K., Zhu Y., Leng S., He Y., Maharjan S., Zhang Y. (2019). Deep Learning Empowered Task Offloading for Mobile Edge Computing in Urban Informatics. IEEE Internet Things J..

[30] Yu X., Guan M., Liao M., Fan X. (2019). Pre-Migration of Vehicle to Network Services Based on Priority in Mobile Edge Computing. IEEE Access.

[31] Lehr H., Chapin M. (2010). On the Convergence of Wired and Wireless Access Network Architectures. Inf. Econ. Policy.

[32] Zhou Z., Liu P., Feng J., Zhang Y., Mumtaz S., Rodriguez J. (2019). Computation Resource Allocation and Task Assignment Optimization in Vehicular Fog Computing: A Contract-Matching Approach. IEEE Trans. Veh. Technol..

[33] Hou X., Li Y., Chen M., Wu D., Jin D., Chen S. (2016). Vehicular Fog Computing: A Viewpoint of Vehicles as the Infrastructures. IEEE Trans. Veh. Technol..

[34] Karedal J., Czink N., Paier A. (2011). Path Loss Modeling for Vehicle-to-Vehicle Communications. IEEE Trans. Veh. Technol..

[35] Lyu X., Tian H., Zhang P. (2016). Multi-User Joint Task Offloading and Resources Optimization in Proximate Clouds. IEEE Trans. Veh. Technol..

[36] Zhang K. (2016). Energy-Efficient Offloading for Mobile Edge Computing in 5G Heterogeneous Networks. IEEE Access.

[37] Wang F., Xu J., Wang X., Cui S. (2018). Joint Offloading and Computing Optimization in Wireless Powered Mobile-Edge Computing Systems. IEEE Trans. Wirel. Commun..

[38] Wei F., Chen S., Zou W. (2018). A greedy algorithm for task offloading in mobile edge computing system. China Commun..

[39] Sun Y. (2019). Adaptive Learning-Based Task Offloading for Vehicular Edge Computing Systems. IEEE Trans. Veh. Technol..

[40] Liu Y., Wang S., Huang J., Yang F. A Computation Offloading Algorithm Based on Game Theory for Vehicular Edge Networks. Proceedings of the IEEE International Conference on Communications (ICC).

[41] Mazza D., Tarchi D., Corazza G. A partial offloading technique for wireless mobile cloud computing in smart cities. Proceedings of the European Conference on Networks and Communications (EuCNC).

